# Microbiome and response surface methodology analyses reveal *Acetobacter pasteurianus* as the core bacteria responsible for aerobic spoilage of corn silage (*Zea mays*) in hot and humid areas

**DOI:** 10.3389/fmicb.2024.1473238

**Published:** 2024-09-11

**Authors:** Rui Bai, Haiping Li, Shiyong Chen, Xianjun Yuan, Youjun Chen, Yanling Huang, Qingping Zhou, Hao Guan

**Affiliations:** ^1^Sichuan Zoige Alpine Wetland Ecosystem National Observation and Research Station, Southwest Minzu University, Chengdu, China; ^2^College of Grassland Resources, Southwest Minzu University, Chengdu, China; ^3^College of Animal Science and Veterinary Medicine, Southwest Minzu University, Chengdu, China; ^4^School of Mathematics and Statistics, Qinghai Normal University, Xining, China; ^5^College of Agro-grassland Science, Nanjing Agricultural University, Nanjing, China

**Keywords:** *Acetobacter pasteurianus*, whole-plant corn silage, aerobic stability, bacterial community, response surface methodology

## Abstract

**Introduction:**

Weak aerobic stability is a notable challenge for whole-plant corn silage, particularly in hot and humid regions. *Acetobacter* is commonly regarded as an indicator of aerobic deterioration in silage, yet its precise role in fermentation and during aerobic exposure, as well as the factors that promote its growth, remain insufficiently understood.

**Methods:**

In this study, whole-plant corn silage was prepared using a bagged method with controlled dry matter (DM) content at 20%, 25%, and 30%, and initial concentrations of *A. pasteurianus* at 40%, 50%, and 60%. The silage was stored for 60 days under varying temperatures (20°C, 30°C, and 40°C). Following the anaerobic storage phase, the silage was exposed to air at room temperature (20-25°C) for 7 days, both with and without *A. pasteurianus* inoculation.

**Results:**

The results demonstrated that *A. pasteurianus* did not impact the nutritional value of the silage during anaerobic fermentation, maintaining a low pH (< 3.80). However, during aerobic exposure, the presence of *A. pasteurianus* significantly reduced the aerobic stability of the silage. The microbial community shifted from primarily *Klebsiella* species initially to *Lactobacillus* and *Acetobacter* species post-ensiling. During the aerobic exposure phase, *A. pasteurianus* and *A. fabarum* became the dominant species. Response Surface Methodology (RSM) analysis identified optimal conditions for the proliferation of *A. pasteurianus* during the aerobic phase, which occurred at 28°C, 25% DM, and 52% initial concentration at 3 ml/kg.

**Discussion:**

These findings confirm that *A. pasteurianus* plays a critical role in reducing the aerobic stability of whole-plant corn silage. Additionally, the study identifies the optimal conditions that favor the proliferation of *A. pasteurianus*, offering valuable insights for the development of strategies to prevent and control this bacterium, thereby improving the aerobic stability of silage in hot and humid regions.

## 1 Introduction

The defining characteristics of hot and humid climatic zones include elevated temperatures, high humidity, substantial precipitation, minimal daily temperature variations, and negligible wind speeds. These conditions are primarily associated with tropical rainforest climates. Currently, an increasing number of dairy farms are undergoing a transition from grazing to confinement systems, including free-stall barns and compost barns. The elevated demand for beef, lamb, and dairy products has resulted in a considerable deficit in the forage required by the livestock industry, thereby necessitating the year-round provision of preserved feed for dairy farms (Miguel et al., 2023). Silage has historically constituted a primary preserved feedstuff, particularly given the growth of forage during the humid, rainy season. It is therefore crucial for farmers to produce high-quality, long-lasting silage on farms during periods of high grain prices to ensure the success of their operations (Daniel et al., 2019).

The conversion of forages with high temperatures, low dry matter (DM) content, and low soluble carbohydrates into high-quality silage was a challenging process due to their high biochemical oxygen demand in tropical and subtropical regions (Loures et al., 2005). Furthermore, the fermentation quality of this type of silage was often unstable, frequently exhibiting high acetic acid fermentation with a noticeable acetic acid smell (Guan et al., 2020). The ensiling of tropical grass may result in the production of large amounts of acetic acid instead of lactic acid (Nishino et al., 2012), despite the initial detection of lactic acid during the ensiling process. However, in subsequent stages, there was often a decline in lactic acid levels and an increase in acetic acid levels (Hou and Nishino, 2021). The fermentation of high acetic acid silage, which was similar to lactic acid fermentation dominated by homofermentative lactic acid bacteria (LAB), often results in a low pH, which can lead to misinterpretation of the quality of the silage fermentation (Kung et al., 2018). It has been documented that silage with a low DM content (less than 20%) can exhibit acetic acid levels as high as 3–6% DM, excessive acetic acid fermentation (greater than 3% DM) has been observed to results in significant DM loss, accelerate aerobic spoilage, and markedly reduce animal intake (Gerlach et al., 2020). It is therefore recommended that high acetic acid fermentation be given particular attention in the production of silage in tropical and subtropical regions.

The process of acetic acid fermentation was primarily driven by heterofermentative LAB, enterobacteria, and *Acetobacter* bacteria (Yan et al., 2022). In particular, during the opening stage of silage in the hot and humid regions, a significant presence of *Acetobacter* bacteria has been observed (Guan et al., 2018). In a study conducted by Carvalho-Estrada et al. (2020), it was reported that after 120 days of fermentation, the abundance of *Acetobacter* bacteria in two varieties of corn silage in São Paulo, Brazil, exceeded 80%. Thi Minh Tu et al. (2014) documented the presence of *Acetobacter* bacteria in small-scale TMR silages in a laboratory setting in Vietnam. *Acetobacter* bacteria were frequently regarded as the primary initiators of aerobic spoilage in silage (Spoelstra et al., 1988). However, their metabolic byproducts were also acetic acid, analogous to the extensively utilized heterofermentative lactic acid bacterium *Lactobacillus buchneri.* The role and mechanism of *Acetobacter* bacteria during the entire silage fermentation and aerobic exposure stages remained unclear.

To investigate the impact of environmental temperature and DM content on the microbial community, fermentation quality, and aerobic stability of silage inoculated with *A. pasteurianus*, whole-plant corn was employed in this study. The application of response surface methodology (RSM) facilitated the identification of the critical points of key factors affecting *A. pasteurianus* proliferation, which will provide a scientific basis for the future prevention and control of *A. pasteurianus* to improve aerobic stability in hot and humid areas.

## 2 Materials and methods

### 2.1 Experimental materials and experimental design

The experiment was conducted at the National Grass Variety Regional Test Base of the Grassland Technology Research and Promotion Center in Sichuan Province, China (N30°76′, E103°76′) in August 2023. The mean maximum temperature was 31°C, with a minimum temperature of 23°C. Please refer to Supplementary Table S1 for further details.

Response Surface Methodology (RSM) is a statistical method employed for the optimization of biological processes (Wang et al., 2016). This method can be employed for the development of models, the evaluation of factors, and the identification of optimal conditions for optimal responses. In our study, RSM was used to examine the influence of environmental factors on the quality of silage: (1) storage temperature (T); (2) DM content (M); (3) initial *A. pasteurianus* concentration (A). The response was the concentration of *A. pasteurianus* produced during the process. The experimental design comprised three levels of each variable: initial *A. pasteurianus* concentration (40, 50, 60%), DM content (30, 25, 20%), and storage temperature (20°C, 30°C, 40°C). RSM was employed to optimize and evaluate the principal interactions and secondary effects of the variables. The variables were arranged according to the Box–Behnken design, which required 17 experiments, including 5 repetitions at the central point (Table 1).

**Table 1:** Group information.

Group	T (°C)	M (%)	A (%)
1 (T30M75A50)	30	25	50
2 (T20M80A50)	20	20	50
3 (T30M75A50)	30	25	50
4 (T30M70A60)	30	30	60
5 (T30M75A50)	30	25	50
6 (T20M75A60)	20	25	60
7 (T20M70A50)	20	30	50
8 (T30M80A40)	30	20	40
9 (T40M80A50)	40	20	50
10 (T40M70A50)	40	30	50
11 (T40M75A40)	40	25	40
12 (T40M75A60)	40	25	60
13 (T20M75A40)	20	25	40
14 (T30M75A50)	30	25	50
15 (T30M70A40)	30	30	40
16 (T30M80A60)	30	20	60
17 (T30M75A50)	30	25	50

T, temperature; M, dry matter content; A, initial concentration of *A. pasteurianus*.

Using Design Expert software (Version 11), the obtained data were analyzed through Analysis of Variance (ANOVA) to evaluate the significance of the model. A second-order polynomial model was used to analyze the experimental data as follows:

$$\gamma =\beta_{0}+\sum \beta_{i}\;X_{j}+\sum \beta_{ii}\;X_{i2}+\sum \beta_{ij}\;X_{i}X_{j}$$

where the predicted response is represented by γ, and X_i_ and X_j_ represent different variables. The intercept, linear, quadratic, and interaction regression coefficients are represented by ß_0_, ß_i_, ß_ii_, and ß_ij_, respectively.

The materials used in this experiment were whole corn plants (*Zea mays* L.), of the variety AoYu 005, with a single field plot area of 15 m^2^ (3 m × 5 m), planted in 3 plots. The whole corn plants were harvested when it reaches the 1/2 milk-line stage on August 3, 2023, with a cutting height of 15 ~ 30 cm above the ground. The processed grain achieved a crushing degree of 80%. The *A. pasteurianus* (CGMCC No. 1.1810) strain was obtained from the China General Microbiological Culture Collection Center (CGMCC).

Whole-plant corn raw materials (RAW) from the 3 plots were harvested separately and chopped into 1–2 cm pieces using a forage cutter (Lingong Machinery, Shandong, China). The maize was ensiled following the same method for each plot, with each batch of silage material weighing 1 kg. The DM content of the silage was adjusted to 30% (M30), 25% (M25), and 20% (M20) as described by Zhu (2021). *A. pasteurianus* solutions at concentrations of 40% (A40), 50% (A50), and 60% (A60) were sprayed evenly onto the silage material at 3 mL/kg (Xu et al., 2019) (initial concentration of 10^6^ cfu·ml^−1^, by mixing 40 mL of a 100% concentration *A. pasteurianus* solution with 60 mL of sterile distilled water to obtain 100 mL of a 40% concentration *A. pasteurianus* suspension. Similarly, prepare *A. pasteurianus* suspension with concentrations of 50 and 60%.). After thorough mixing, each batch of silage material was packed into polyethylene plastic bags (30 cm × 40 cm), vacuum-sealed using a vacuum sealer (DZQ-390, Anshengke, Fujian, China), and stored in temperature-controlled incubators at 20°C (T20), 30°C (T30), and 40°C (T40) for 60 days. Each treatment had 3 replicates. Subsequently, the 60-day silage was subjected to an aerobic stability test, including treatments with *A. pasteurianus* (ASA) and controls without *A. pasteurianus* (ASN), using the same methods as before ensiling. Each sample weighed 300 g, with 3 replicates for each treatment. For each experimental unit, 800 g of silage was packed in a 1 L container compacted, placed in an incubation room at 20°C, and covered with 2 layers of cheesecloth. A temperature probe was placed in the center of the silage and connected to a data logger (BCL3016P multipoint temperature recorder, A-BF). An additional temperature probe was placed in an empty bin to record the ambient temperature as a control. Spoiled samples were collected immediately and unspoiled samples were collected at the end of the 7-day trial period.

### 2.2 Fermentation quality

Samples were thoroughly mixed, and 20 g of fresh material (FM) was accurately weighed and placed in 15 × 20 cm polyethylene plastic bags. 180 mL of distilled water was added, and the samples were homogenized and sealed, then extracted overnight at 4°C (Jiang et al., 2023). The plant residue was filtered through four layers of cheesecloth, and the pH of the filtrate was measured immediately. The remaining filtrate was stored at −20°C for the determination of organic acids and ammonia nitrogen (NH_3_-N) content. All samples were stored at −80°C for further DNA extraction and sequencing analysis.

The pH was measured using a glass electrode pH meter (PHSJ-5; LEICI, Shanghai, China); the NH_3_-N content was determined using the phenol-hypochlorite colorimetric method (Broderick and Kang, 1980); the filtrate was filtered through a 0.22 μm filter and analyzed for lactic acid (LA), acetic acid (AA), propionic acid (PA), and butyric acid (BA) concentration using an ultra-high performance liquid chromatograph (Thermo Fisher UltiMate3000, United States). The chromatographic conditions were an RSpak KC-811 column, 0.1% H_3_PO_4_ as the mobile phase, a flow rate of 0.5 mL·min^−1^, a column temperature of 55°C, and a detection wavelength of 210 nm (Zhao et al., 2016).

### 2.3 Nutritional quality

Fresh material and silage samples were inactivated in a ventilated oven at 105°C for 15 min, then dried in a ventilated oven at 65°C for 48 h to a constant weight to partially dry the material prior to grinding (Zhao et al., 2021). Samples were pulverized into fine powder using a grinder (CT293 CyclotecTM, FOSS Analytical A/S, Hillerød, Denmark), passed through a 1 mm sieve for subsequent experiments.

The DM and ash content of the fresh material and silage were analyzed following the standard methods set forth by the Association of Official Analytical Chemists (AOAC). The crude protein (CP) content was determined utilizing a KjeltecTM8400 automatic Kjeldahl nitrogen analyzer (AOAC, 1990). The neutral detergent fiber (NDF, without heat-stable amylase and including residual ash) and acid detergent fiber (ADF, including residual ash) were measured using a fiber analyzer (Fibretherm FT12, C. Gerhardt, Germany) as described by Van Soest et al. (1991).

### 2.4 Aerobic stability evaluation

The aerobic stability of the silage was determined by measuring the temperature at the center of the bagged silage following the methodology proposed by Ranjit and Kung (2000). The temperature of the core area of the silage (at a depth of 10 cm) was measured by real-time temperature logging (MT-X, Shenhua Science and Technology Co., Ltd., Shenzhen, China) every 5 min for 7 days, with 3 replicates for each treatment.

### 2.5 DNA extraction

Guan et al. (2018) described the DNA extraction method. A total of 20 g of frozen sample was added to 180 mL of sterile distilled water and incubated at 4°C with agitation for 15 min, followed by centrifugation at 8000 rpm for 10 min at 4°C. The supernatant was removed, and the pellet was retained for DNA extraction using the bacterial DNA extraction kit (DP302-02, Tiangen, Beijing, China). DNA purification was performed using the purification kit (DP214-02, Tiangen, Beijing, China). The purity and concentration of the DNA were assessed with a NanoDrop 2000 before storing the DNA samples at −80°C. Qualified DNA samples were subsequently stored at −20°C.

### 2.6 Microbial analysis based on sequencing

The full-length 16S rRNA genes of the D60, ASA, and ASN groups were amplified by PCR using the primers (5′-AGRGTTTGATYNTGGCTCAG-3′) and (5′-TASGGHTACCTTGTTASGACTT-3′). The primers were supplied by Beijing Biomarker Technologies Co., Ltd. The amplified products were subjected to sequencing on the PacBio Sequel platform (Pacific Biosciences, Menlo Park, CA, United States). The raw subreads were corrected to obtain circular consensus sequencing (CCS) sequences using the SMRT (version 8.0) software. Lima software (version 1.7.0) was employed to identify the CCS sequences from different samples through barcode sequences and to remove chimeras, thereby obtaining high-quality CCS sequences. Operational taxonomic unit (OTU) clustering was conducted at a similarity level of 97% using USEARCH (version 10.0). A threshold of 0.005% of all sequences was used to filter OTUs by default. The DADA2 method in QIIME2 (version 2020.6) was used to denoise the quality-controlled data to obtain the optimal sequences.

### 2.7 Absolute quantification of *Acetobacter pasteurianus*

The method of Li et al. (2020) was employed to quantify Acetobacter bacteria DNA using real-time fluorescence quantitative polymerase chain reaction (RT-FQPCR). Universal primers for *A. pasteurianus* (5′-AAGGGGGCTAGCGTTGCTCG-3′ and 5′-ACCGCCTACACGCCCTTTACG-3′) were used. A standard curve of the target *A. pasteurianus* DNA concentration versus the cycle threshold (Ct) value was constructed through serial gradient dilutions, and a melting curve was generated. Subsequently, the DNA extraction products from the D60, ASA, and ASN groups underwent amplification by PCR, and the Ct values were compared with the standard curve to quantify *A. pasteurianus*. The RT-PCR reaction procedure was as follows: stage 1—initial denaturation at 95°C for 5 min; stage 2—95°C for 10 s, 60°C for 30 s, for a total of 40 cycles; stage 3—the instrument’s default melting curve.

### 2.8 Statistical analysis

SPSS software was employed for statistical analysis with one-way ANOVA and Duncan’s multiple-range test. The GraphPad Prism (version 10.0) software was employed for plotting. The absolute quantitative results for *A. pasteurianus* were subjected to analysis using Design-expert (version 11.0). All values are the mean of 3 replicates.

## 3 Results

### 3.1 Chemical compositions of forage and silage

As illustrated in Table 2, following the processing of fresh materials, the DM was 33.41% of the original weight, and the contents of CP, NDF, ADF, starch, and ash were 8.92% DM, 51.63% DM, 21.73% DM, 29.85% DM, and 4.01% DM, respectively. Following a 60-day ensiling period, the CP content ranged from 8.17% DM to 8.84% DM, which exhibited a significant reduction in comparison to the raw material (*p* < 0.05). The NDF content showed no significant differences among the groups, and it was significantly diminished in comparison to the raw material (*p* < 0.05). The ADF and starch contents showed no significant differences and no significant divergence from the raw material. The ash content varied between 4 and 5.5% DM.

**Table 2:** Chemical composition of forage and silages.

Items	DM (%)	CP (% DM)	NDF (% DM)	ADF (% DM)	Starch (% DM)	Ash (% DM)
Forage	33.41^a^	8.92^a^	51.63^a^	21.73	29.85	4.01^m^
1 (T30M75A50)	32.97^b^	8.45^def^	48.39^b^	21.56	28.83	4.69^d^
2 (T20M80A50)	32.98^b^	8.49^cdef^	47.63^b^	21.23	28.98	4.40^ij^
3 (T30M75A50)	32.87^b^	8.76^ab^	48.41^b^	21.55	29.06	4.66^de^
4 (T30M70A60)	32.85^b^	8.67^abcd^	47.28^b^	20.81	29.17	4.68^d^
5 (T30M75A50)	33.01^b^	8.73^abc^	48.36^b^	21.3	28.85	5.22^b^
6 (T20M75A60)	32.95^b^	8.50^cdef^	47.37^b^	20.66	29.12	5.09^c^
7 (T20M70A50)	32.92^b^	8.30^fg^	47.19^b^	21.07	29.00	4.37^k^
8 (T30M80A40)	32.90^b^	8.17^g^	47.59^b^	21.32	28.67	4.37^k^
9 (T40M80A50)	32.70^b^	8.17^g^	48.58^b^	21.2	29.4	4.48^hi^
10 (T40M70A50)	32.74^b^	8.84^ab^	47.77^b^	21.08	28.77	4.59^efg^
11 (T40M75A40)	32.74^b^	8.34^fg^	47.67^b^	21.28	28.38	4.21^l^
12 (T40M75A60)	32.72^b^	8.48^def^	47.54^b^	20.58	29.24	5.05^c^
13 (T20M75A40)	32.80^b^	8.48^def^	48.68^b^	21.33	28.86	4.51^gh^
14 (T30M75A50)	32.81^b^	8.82^ab^	47.83^b^	21.64	29.44	5.42^a^
15 (T30M70A40)	32.91^b^	8.46^def^	47.55^b^	20.06	28.81	4.41^ij^
16 (T30M80A60)	32.70^b^	8.37^efg^	47.20^b^	20.32	28.66	4.56^fgh^
17 (T30M75A50)	32.77^b^	8.61^bcde^	48.74^b^	21.21	28.63	4.64^def^
SEM	0.176	0.107	0.786	0.741	0.614	0.042
P	0.065	<0.001	<0.001	0.722	0.055	<0.001

DM, dry matter; CP, crude protein; NDF, neutral detergent fiber; ADF, acid detergent fiber. Values with different superscripts in the same column indicate significant differences (*p* < 0.05).

### 3.2 Fermentation quality on silages inoculated with *Acetobacter pasteurianus*

The final fermentation products of the silage samples and aerobic exposure samples were shown in Table 3. The pH of the D60 group samples was below 3.80, with the lowest reading being 3.46 in group 16. During the transition from the fermentation phase to the aerobic exposure phase, a tendency for the pH and NH_3_-N content to increase. Group 6 exhibited the lowest NH_3_-N content (20.38 mg dL^−1^), with LA content ranging from 3.04% DM to 4.84% DM, AA content varying between 0.73% DM and 1.84% DM, and no PA and BA detected. In the ASA group samples, the pH exhibited a range of 4.12–6.21, while the NH_3_-N content ranged from 286.83 to 316.31 mg dL^−1^. Notably, LA was undetected in groups 2, 4, 6, 8, and 9, while in other groups, it varied between 0.43% DM and 3.14% DM. Similarly, AA content ranged from 0.25% DM to 1.39% DM across all groups. Additionally, PA was identified in groups 2, 4, 6, 7, 9, 10, 13, and 16, with concentrations ranging from 0.22% DM to 0.78% DM. No BA was detected in any of the samples. In the ASN group samples, the pH exhibited a range of 3.84 to 4.91, while the NH_3_-N content spanned from 217.98 to 254.07 mg dL^−1^. The LA content varied between 0.68% DM and 6.52% DM, and the AA content ranged from 0.54% DM to 2.07% DM. Notably, no PA or BA was detected in these samples.

**Table 3:** Changes in fermentation quality during silage fermentation to aerobic exposure stages.

Items	pH			NH_3_-N (mg·dL^−1^)			LA (%DM)			AA (%DM)			PA (%DM)			BA (%DM)		
	D60	ASA	ASN	D60	ASA	ASN	D60	ASA	ASN	D60	ASA	ASN	D60	ASA	ASN	D60	ASA	ASN
1 (T30M75A50)	3.64^cd^	4.21^ij^	4.31^b^	24.92^abc^	292.92^ghi^	226.51^ef^	4.28^bc^	3.09^a^	3.11^f^	1.26^bc^	1.2^b^	1.43^bc^	ND	ND	ND	ND	ND	ND
2 (T20M80A50)	3.48^hi^	5.62^cd^	4.09^d^	22.39^defg^	311.16^abc^	234.01^bcdef^	4.28^bc^	ND	2.05^i^	1.08^cde^	0.37^hij^	0.91^ef^	ND	0.3^cd^	ND	ND	ND	ND
3 (T30M75A50)	3.71^bc^	5.56^d^	4.30^b^	26.34^a^	305.01^bcdef^	243.08^abcde^	3.97^ef^	1.30^d^	2.2^hi^	1.07^cde^	0.73^ef^	1.21^cde^	ND	ND	ND	ND	ND	ND
4 (T30M70A60)	3.56^efgh^	5.66^c^	3.90^fg^	24.17^abcd^	295.73^fghi^	230.24^cdef^	4.84^a^	ND	6.52^a^	1.42^b^	1.39^a^	1.54^b^	ND	0.78^ab^	ND	ND	ND	ND
5 (T30M75A50)	3.61^de^	4.12^j^	4.32^b^	25.59^ab^	307.2^abcde^	226.85^ef^	4.07^cde^	1.75^c^	2.37^gh^	1.12^cd^	0.94^cd^	1.23^bcd^	ND	ND	ND	ND	ND	ND
6 (T20M75A60)	3.50^hi^	6.21^a^	3.88^fg^	20.38^g^	316.18^a^	217.98^f^	3.14^ghi^	ND	3.77^e^	0.78^gh^	0.93^cd^	1.14^cde^	ND	0.24^cd^	ND	ND	ND	ND
7 (T20M70A50)	3.53^efghi^	4.77^f^	3.86^g^	22.3^defg^	316.31^a^	219.56^f^	4.35^b^	1.13^d^	3.11^f^	1.13^cd^	0.47^ghi^	1.14^cde^	ND	0.34^c^	ND	ND	ND	ND
8 (T30M80A40)	3.51^ghi^	6.15^a^	4.91^a^	20.48^g^	309.85^abcd^	244.2^abcde^	2.92^i^	ND	0.68^j^	0.73^h^	0.65^fg^	0.65^fg^	ND	ND	ND	ND	ND	ND
9 (T40M80A50)	3.56^efgh^	6.14^a^	3.86^g^	20.15^g^	299.57^defgh^	250.84^ab^	3.13^ghi^	ND	4.91^c^	0.74^h^	0.28^ij^	1.2^cde^	ND	0.5^bc^	ND	ND	ND	ND
10 (T40M70A50)	3.76^ab^	4.52^g^	4.18^c^	23.86^bcde^	286.83^i^	247.39^abc^	3.04^hi^	1.57^c^	3.15^f^	1.84^a^	0.87^de^	2.07^a^	ND	0.9^a^	ND	ND	ND	ND
11 (T40M75A40)	3.60^def^	4.29^hi^	3.90^fg^	22.96^cdef^	304.75^bcdef^	254.07^a^	3.24^gh^	1.75^c^	3.96^e^	0.89^efgh^	0.65^fg^	1.23^bcd^	ND	ND	ND	ND	ND	ND
12 (T40M75A60)	3.64^cd^	4.32^h^	3.95^ef^	21.8^efg^	309.26^abcde^	246.51^abcd^	3.75^f^	2.17^b^	4.33^d^	1.02^def^	0.48^gh^	1.24^bcd^	ND	ND	ND	ND	ND	ND
13 (T20M75A40)	3.52^fghi^	5.42^e^	4.00^e^	21.25^fg^	291.14^hi^	222.4^f^	3.34^g^	0.72^e^	3.17^f^	0.86^fgh^	0.25^j^	0.95^de^	ND	0.22^cd^	ND	ND	ND	ND
14 (T30M75A50)	3.59^defg^	4.30^hi^	4.28^b^	25.38^ab^	302.04^cdefg^	227.56^def^	4.04^de^	2.14^b^	2.54^g^	1.13^cd^	1.06^bcd^	1.29^bc^	ND	ND	ND	ND	ND	ND
15 (T30M70A40)	3.54^efghi^	5.76^b^	3.84^g^	21.56^efg^	313.45^ab^	243.26^abcde^	3.81^f^	0.43^f^	5.24^b^	0.91^efgh^	0.72^ef^	1.45^bc^	ND	ND	ND	ND	ND	ND
16 (T30M80A60)	3.46^i^	5.33^e^	4.14^cd^	20.85^fg^	298.39^efgh^	242.41^abcde^	3.32^g^	0.84^e^	2.64^g^	0.96^defg^	0.41^hij^	0.54^g^	ND	0.48^bc^	ND	ND	ND	ND
17 (T30M75A50)	3.80^a^	4.27^hi^	4.32^b^	25.79^ab^	301.69^cdefg^	247.45^abc^	4.22^bcd^	3.14^a^	3.08^f^	1.21^c^	1.12^bc^	1.41^bc^	ND	ND	ND	ND	ND	ND
SEM	0.0355	0.0453	0.0351	1.02614	4.7048	8.2729	0.103	0.112	0.145	0.085	0.090	0.133		0.140				
*P*	<0.001	<0.001	<0.001	<0.001	<0.001	<0.001	<0.01	<0.01	<0.01	<0.01	<0.01	<0.01		<0.001				

D60, whole-plant corn ensiled for 60 days; ASA, silages inoculated with *A. pasteurianus* during aerobic exposure; ASN, silages without *A. pasteurianus* during aerobic exposure. LA, lactic acid; AA, acetic acid; PA, propionic acid; BA, butyric acid. Values with different superscripts in the same column indicate significant differences (*P* < 0.05).

### 3.3 Aerobic stability of silage

**Figure 1:**
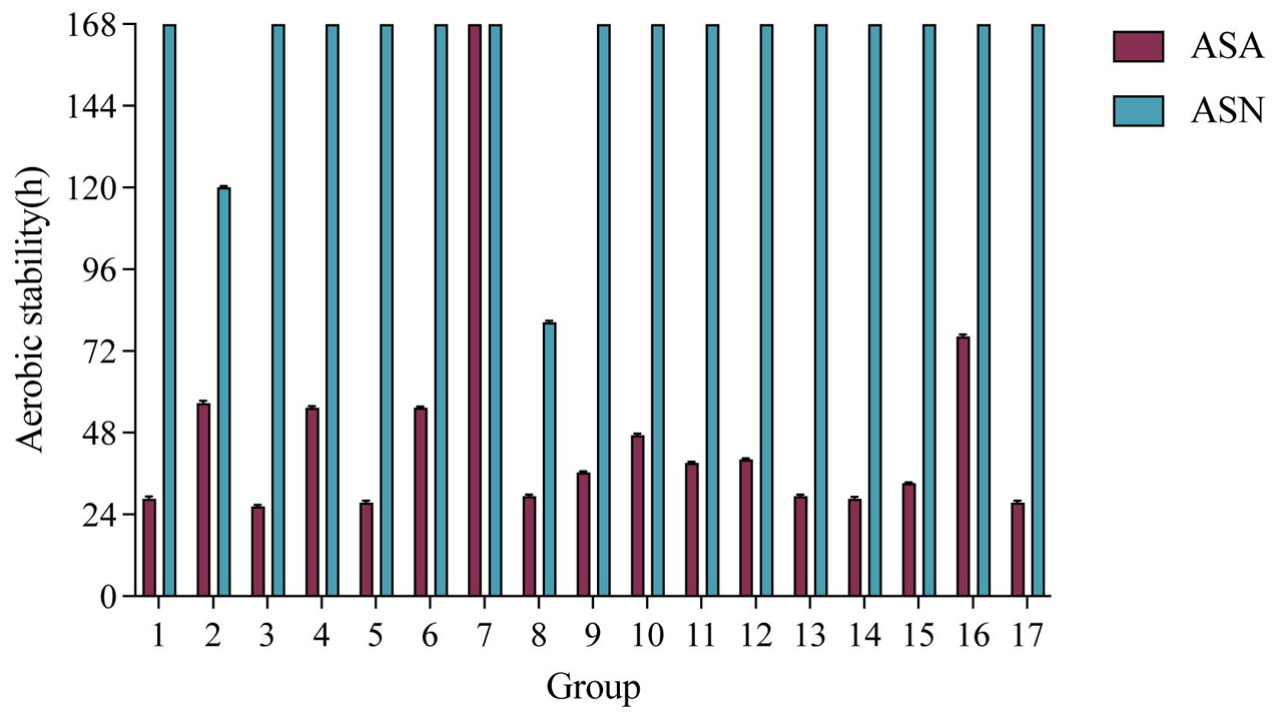
Effect of *A. pasteurianus* on the aerobic stability of whole-plant corn silage after 60 days of fermentation. ASA, silages inoculated with *A. pasteurianus* during aerobic exposure; ASN, silages without *A. pasteurianus* during aerobic exposure.

Figure 1 illustrates the duration of aerobic stability. With the prolongation of exposure time, the temperature of each treatment group exhibited a gradual increase. The ASN group demonstrated the greatest overall stability, with the center temperature of the majority of silage samples remaining below the ambient temperature by 2°C after 7 days. Group 2 and Group 8 exhibited signs of spoilage at 120 and 80 h of aerobic exposure, respectively. The ASA group exhibited a tendency towards rapid spoilage, with the majority of samples showing signs of deterioration within a period of 48–120 h. It is noteworthy that Group 7 in the ASA group exhibited the longest aerobic stability duration, exceeding 168 h.

### 3.4 Multifactorial interaction of absolute quantification of *Acetobacter pasteurianus* under different environmental conditions

The three independent factors selected for RSM optimization were temperature, DM content, and initial concentration of *A. pasteurianus*. The Box–Behnken design (BBD) yielded a total of 17 sets of experiments, the results of which are presented in Table 4. The concentration of *A. pasteurianus* obtained from the ASN phase in these experiments ranged from 0.67 to 5.59 log cfu·ml^−1^.

**Table 4:** The Box–Behnken design with independent factors and observed values for the absolute quantitative results of *A. pasteurianus.*

Runs	Level of independent factors			Concentration of *A. pasteurianus* (log cfu·ml^−1^)
	X_1_(°C)	X_2_(%)	X_3_(%)	
1	30	25	50	5.59
2	20	30	50	2.31
3	40	30	50	0.67
4	30	25	50	5.36
5	30	25	50	5.36
6	30	25	50	5.3
7	30	20	60	3.64
8	20	25	40	2.26
9	20	25	60	3.1
10	30	20	40	2.81
11	30	30	60	3.41
12	20	20	50	2.45
13	30	25	50	5.26
14	30	20	40	1.84
15	40	25	40	1.02
16	40	25	60	2.28
17	40	20	50	1.93

X_1_, temperature; X_2_, dry matter content; X_3_, initial concentration of *A. pasteurianus*.

The statistical significance of the second-order response surface model was evaluated through one-way ANOVA, as illustrated in Table 5. The model exhibited a high *F*-value (391.28) and a low *p*-value (<0.001). In contrast, the *F*-value of the model’s lack of fit was 0.7884 (*p* > 0.05), which was not statistically significant. Furthermore, the model exhibited a low coefficient of variation (C.V.% = 3.39), *R*^2^ = 0.9980, and an adjusted *R*^2^ = 0.9955, indicating that this model can explain 99.80% of the variability in the three factors, and 99.55% of *A. pasteurianus* content under environmental conditions can be explained by this model.

**Table 5:** Analysis of variance for absolute quantitative response surface modelling of *A. pasteurianus*.

Source	Sum of squares	df	Mean square	*F*-value	*p*-value	Significance
Model	41.7254	9	4.6362	391.28	<0.0001	**
A-Temperature	2.2261	1	2.2261	187.87	<0.0001	**
B-dry matter	0.8450	1	0.8450	71.32	<0.0001	**
C-Initial concentration	2.5313	1	2.5313	213.63	<0.0001	*
AB	0.3136	1	0.3136	26.47	0.0013	**
AC	0.0441	1	0.0441	3.72	0.0950	NS
BC	0.1369	1	0.1369	11.55	0.0115	*
A^2^	19.4145	1	19.4145	1638.53	<0.0001	**
B^2^	8.1037	1	8.1037	683.93	<0.0001	**
C^2^	4.7516	1	4.7516	401.02	<0.0001	**
Residual	0.0829	7	0.0118			
Lack of fit	0.0175	3	0.0058	0.3566	0.7884	NS
Pure error	0.0654	4	0.0164			
Cor total	41.8083	16				
R^2^	0.9980					
Adjusted R^2^	0.9955					
Predicted R^2^	0.9909					
Adeq precision	55.9033					

*Stands for statical significance (*P* < 0.05), ** Stands for statical extremely significance (*p* < 0.001), NS stands for no significance.

The impact of each factor on the total concentration of *A. pasteurianus* is inversely proportional to the square of the factor’s *F*-value. Therefore, the larger the F value, the greater the impact of the single factor on the total concentration of *A. pasteurianus*. The order of influence of the individual factors was as follows: C (initial *A. pasteurianus* concentration) > A (temperature) > B (DM content). The linear, interaction, and quadratic parameters were all found to be statistically significant. A multiple regression analysis was conducted on the experimental data to derive a second-order polynomial equation that describes the relationship between the variables and the response. The fitting equation parameters are as follows:

$$\begin{array}{l} y=5.3746-0.5275A+0.325B+0.5625C+0.28\mathrm{AB}\\ \;+0.105\;\mathrm{AC}-0.185\;BC-2.15A^{2}-1.39B^{2}-1.06C^{2}\text{.} \end{array}$$

**Figure 2:**
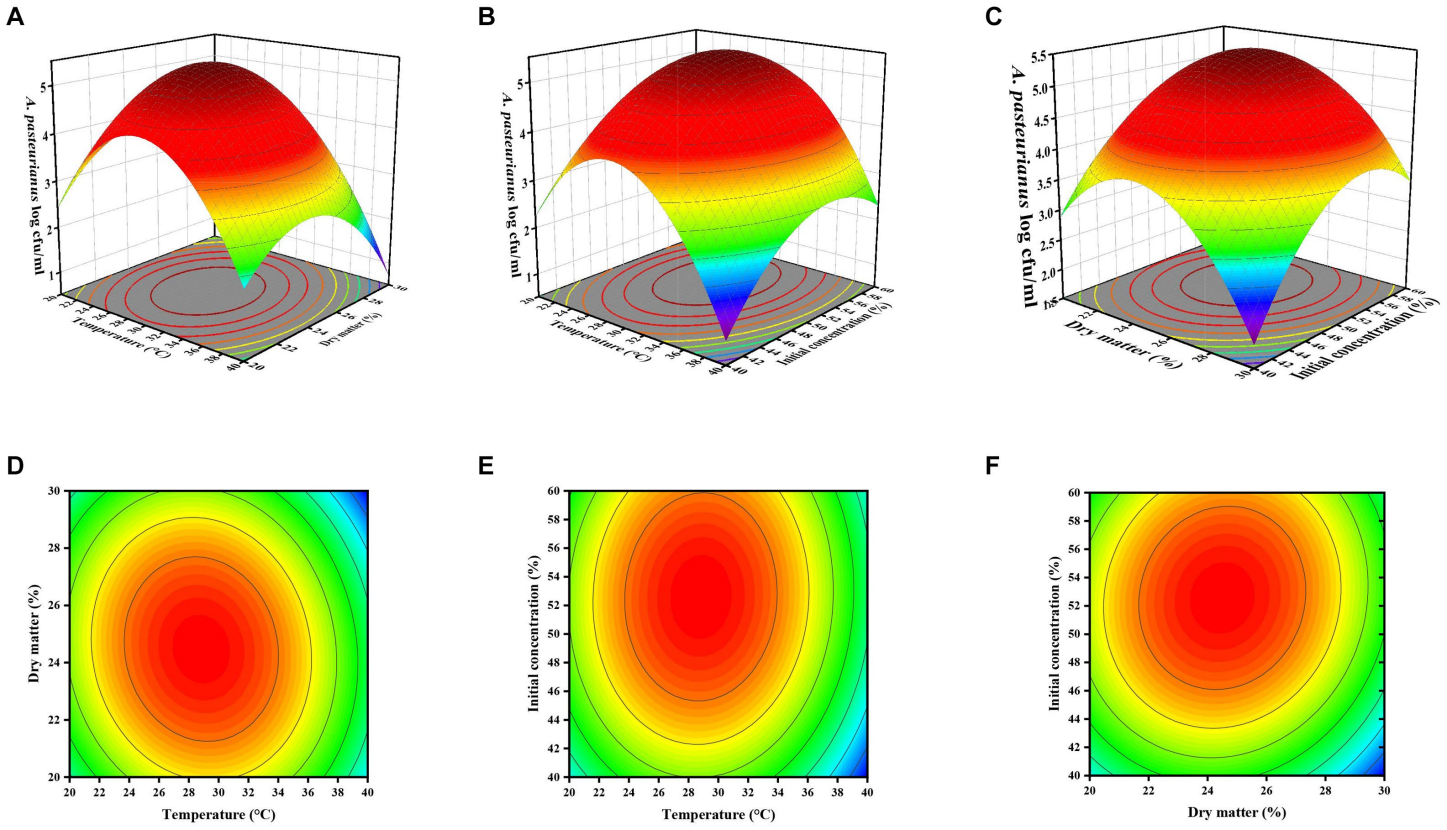
Three-dimensional response surface plots **(A,C,E)** and two-dimensional contour plots **(B,D,F)** of ASN *A. pasteurianus* content **(A–F)**, respectively, represent the interactions between temperature, dry matter content, and initial *A. pasteurianus* concentration.

Three-dimensional (3D) and two-dimensional (2D) response surface and contour plots were employed to predict the interactive effects of temperature, pH, and initial *A. pasteurianus* concentration on the growth of *A. pasteurianus* (Figure 2). As illustrated in the figure, the initial concentration of *A. pasteurianus* in the silage exhibited a more positive impact on the total concentration of *A. pasteurianus* in the ASN group. The results of the ANOVA and the response surface plots indicated that the interactions between storage temperature and DM content (AB) and DM content and initial *A. pasteurianus* concentration (AC) were significant.

Furthermore, under the predicted optimal conditions—temperature of 28.889°C, DM content of 24.557%, and initial *A. pasteurianus* concentration of 52.517%—the ASN stage had the highest *A. pasteurianus* content. At a temperature of 39.853°C, DM content of 29.909%, and initial *A. pasteurianus* concentration of 56.690%, the ASN stage exhibited the lowest *A. pasteurianus* content (Table 6). For the purposes of practical processing, it is necessary to verify the aforementioned conditions to be whole numbers. The data presented in figures a-f illustrate that as temperature and DM content, as well as temperature and initial concentration of *A. pasteurianus*, and DM content and initial concentration of *A. pasteurianus* increase, the *A. pasteurianus* content in the ASN group initially rises before declining. The data presented above demonstrate that environmental temperature and the initial concentration of *A. pasteurianus* are key factors affecting the increase in *A. pasteurianus* concentration during aerobic exposure, which is related to the DM content of the raw materials.

**Table 6:** Predicted value of *A. pasteurianus* content.

Number	Temperature	Dry matter content	Initial *A. pasteurianus* concentration	Absolute quantity	Desirability
1	28.889	24.557	52.517	5.489(Max)	0.979
2	39.853	29.909	56.690	0.667(Min)	1

### 3.5 Dynamics of silage microbial diversity at different stages

**Figure 3:**
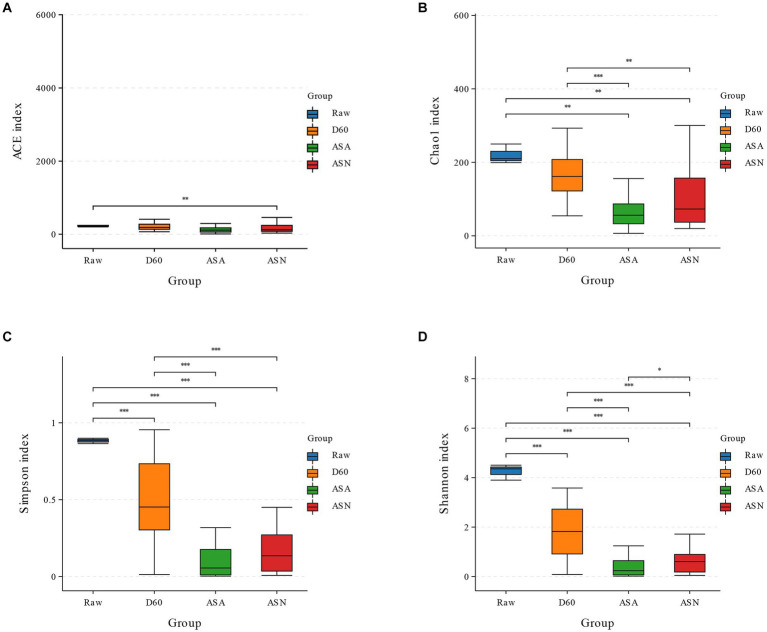
Bacterial alpha diversity of silage (**A**, ACE index; **B**, Chao1 index; **C**, Shannon index; **D**, Simpson index). RAW, pre-ensiled silage; D60, whole-plant corn ensiled for 60 days; ASA, silages inoculated with *A. pasteurianus* during aerobic exposure; ASN, silages without *A. pasteurianus* during aerobic exposure.

Based on SMRT sequencing of the full-length 16S rRNA genes of silage bacteria, an average of 12,814 CCS sequences were obtained from each sample. Alpha diversity, reflecting bacterial richness and diversity, was characterized using the ACE, Chao1, Shannon, and Simpson indices (Figure 3). In this study, the Chao1 index of all samples significantly decreased after silage (*p* < 0.05), while the ACE index decreased but not significantly. The Chao1, ACE, Simpson, and Shannon indices of the ASA and ASN groups were significantly lower than the D60 group (*p* < 0.05). The bacterial richness and community diversity in the ASN group were found to be significantly higher than in the ASA group (*p* < 0.05).

**Figure 4:**
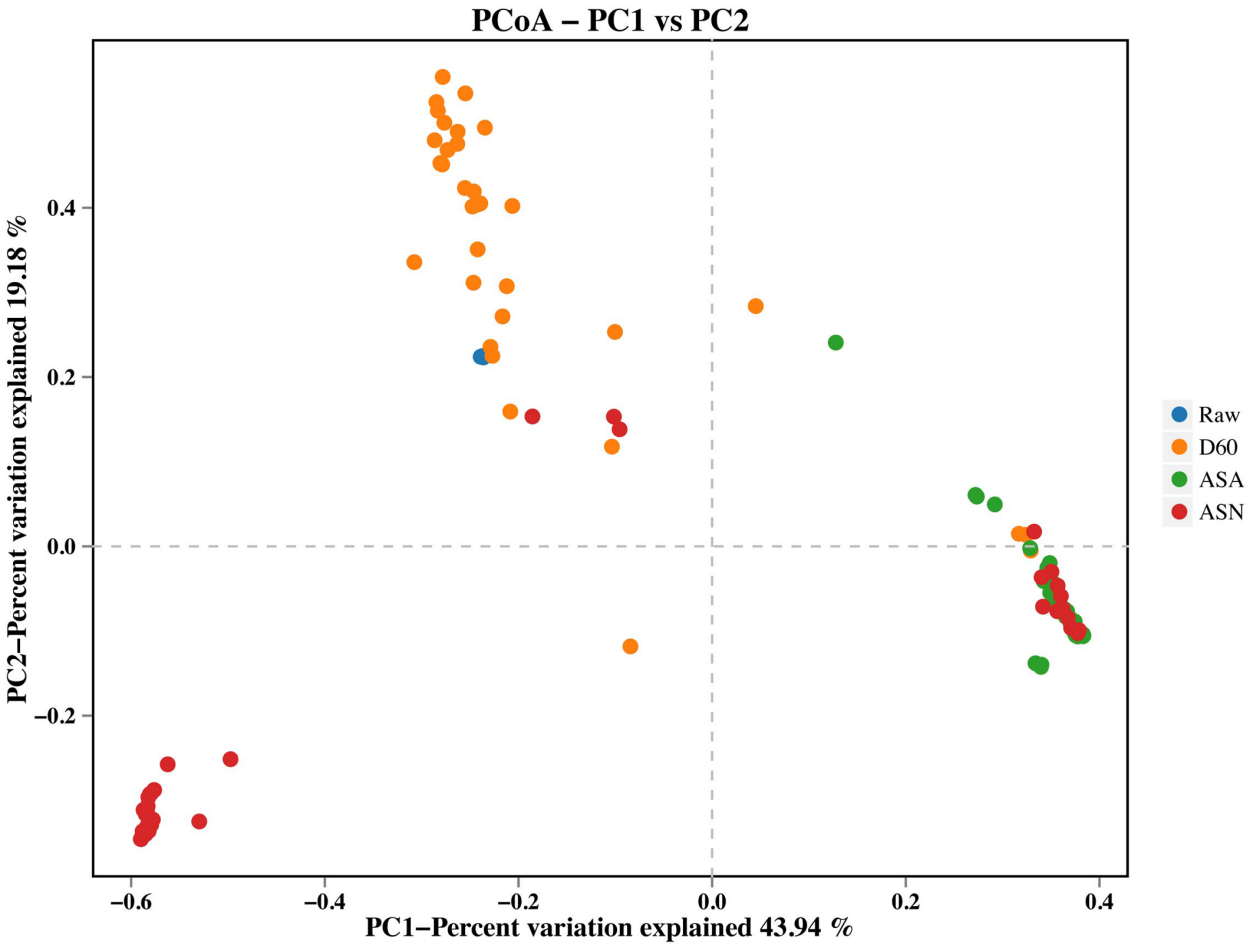
Bacterial beta diversity of silage. RAW, pre-ensiled silage; D60, whole-plant corn ensiled for 60 days; ASA, silages inoculated with *A. pasteurianus* during aerobic exposure; ASN, silages without *A. pasteurianus* during aerobic exposure.

Principal coordinate analysis (PCoA) of beta diversity highlighted differences in bacterial communities across various treatments during the raw material, fermentation, and aerobic exposure stages (Figure 4). The data presented in the figure reveal a significant degree of dispersion between the D60 group and the ASA group, as well as between the D60 group and the ASN group.

**Figure 5:**
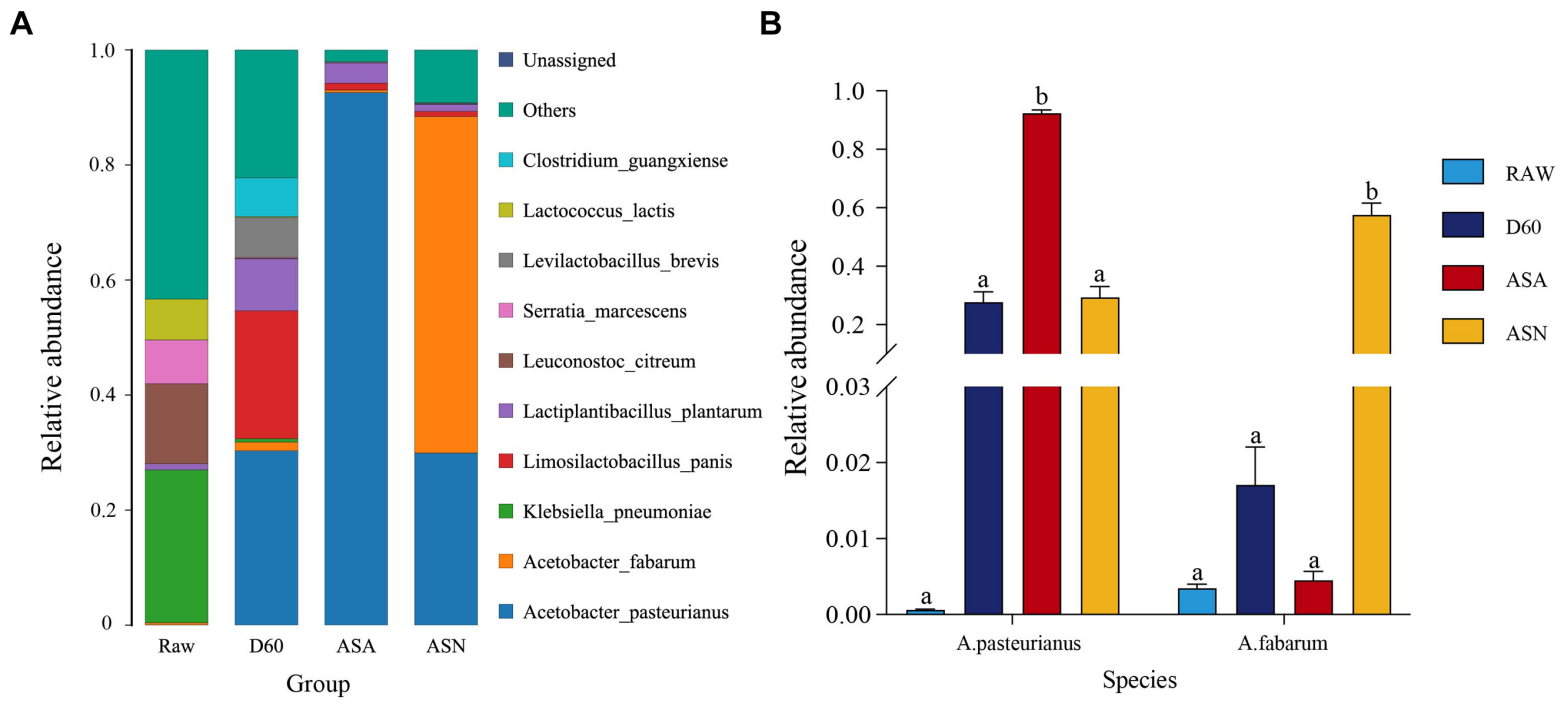
Bacterial community structure at the species level **(A)** and relative abundance of *A. pasteurianus* and *A. fabarum* at the species level **(B)** in pre-ensiled, ensiled, and aerobically exposed silage. RAW, pre-ensiled silage; D60, whole-plant corn ensiled for 60 days; ASA, silages inoculated with *A. pasteurianus* during aerobic exposure; ASN, silages without *A. pasteurianus* during aerobic exposure.

Figure 5A illustrated that at the species level, the epiphytic microbial flora of fresh corn mainly consisted of *Klebsiella pneumoniae* (26.58%), *Leuconostoc citreum* (13.93%), *Serratia marcescens* (7.61%), *Lactiplantibacillus plantarum* (1.06%), and *A. pasteurianus* (0.35%). After 60 days of ensiling, the following microbial species were identified: *A. pasteurianus* (30.31%), *Limosilactobacillus panis* (22.25%), *Lactiplantibacillus plantarum* (9.05%), *Levilactobacillus brevis* (6.94%), *Clostridium guangxiense* (6.75%), and *A. fabarum* (1.52%). The D60 group exhibited an increased relative abundance of *A. pasteurianus*, *Limosilactobacillus panis*, *L. plantarum*, *Levilactobacillus brevis*, *Clostridium guangxiense*, and *A. fabarum*, while the relative abundances of *Serratia marcescens* and *Klebsiella pneumoniae* were reduced. During the aerobic exposure stage, irrespective of the treatment, the *A. pasteurianus* genus was the most prevalent. In the ASN group, the genus *A. fabarum* exhibited the highest relative abundance (58.51%), followed by *A. pasteurianus* (29.94%) and *L. plantarum* (1.22%). In the ASA group, *A. pasteurianus* (92.61%) had the highest relative abundance, accompanied by a minor presence of *L. plantarum* (3.15%). Figure 5B showed that the abundance of *A. pasteurianus* in the ASA group was significantly higher than in the RAW, D60, and ASN groups (*p* < 0.05). Additionally, the abundance of *A. fabarum* in the ASN group is significantly higher compared to the RAW, D60, and ASA groups (*p* < 0.05).

**Figure 6:**
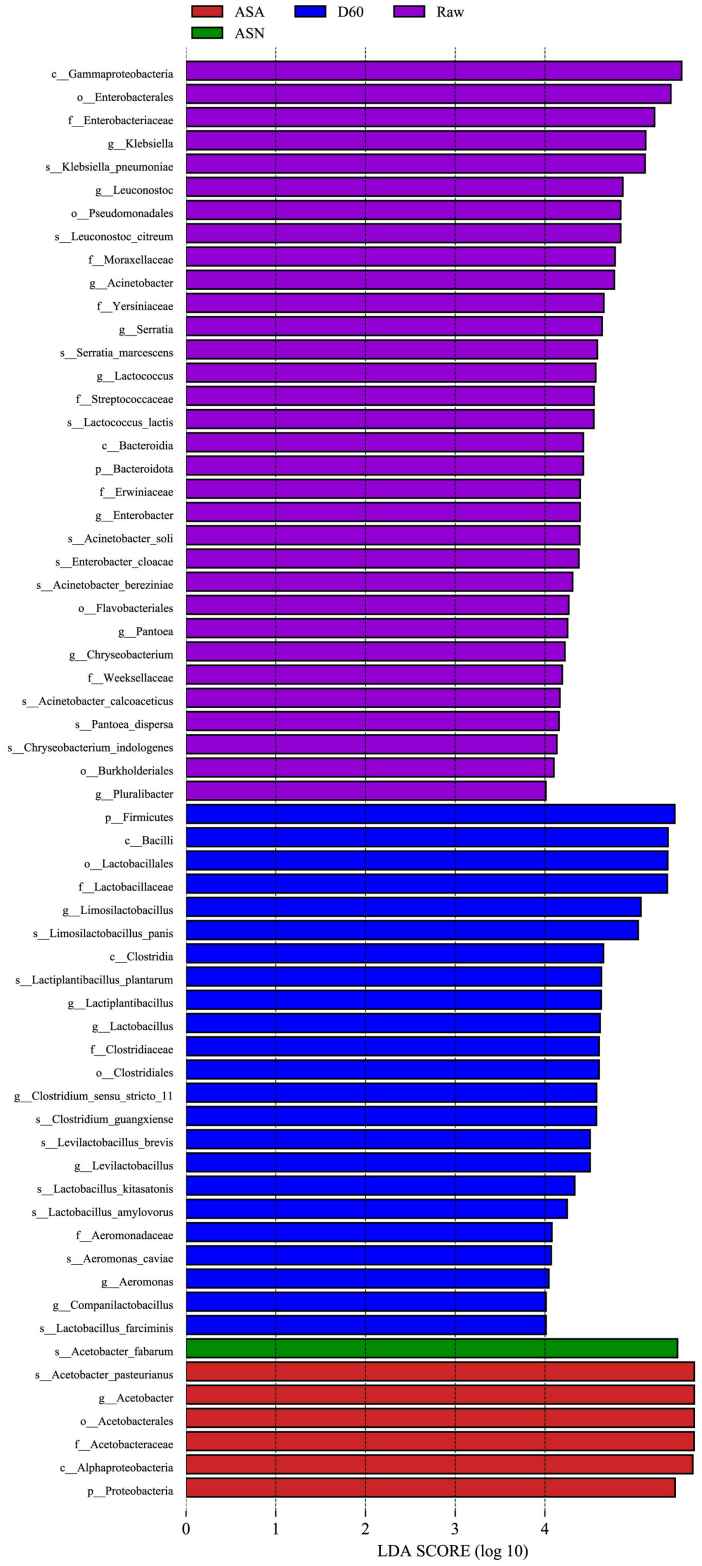
LEfSe analysis of pre-ensiled forage, ensiled forage, and aerobic-exposed ensiled forage. RAW, pre-ensiled silage; D60, whole-plant corn ensiled for 60 days; ASA, silages inoculated with *A. pasteurianus* during aerobic exposure; ASN, silages without *A. pasteurianus* during aerobic exposure.

Linear discriminant analysis effect size (LEfSe, LDA = 4) was used to identify differences in bacterial community structure among the groups (Figure 6). This analysis calculates the relative abundance of bacterial genera in RAW, D60, ASA, and ASN groups. LEfSe identifies the taxa that are most likely to explain the differences in bacterial community structure among the groups. The results indicated that the genera *Klebsiella*, *Leuconostoc*, *Acinetobacter*, *Serratia*, *Lactococcus*, *Enterobacter*, *Pantoea*, *Chryseobacterium*, and *Pluralibacter* are enriched in the RAW group. The genera *Limosilactobacillus*, *Lactiplantibacillus*, *Lactobacillus*, *Clostridium sensu stricto 11*, *Levilactobacillus*, and *Aeromonas* were enriched in the D60 group. The ASA group was characterized by an enrichment of *A. pasteurianus*, while the ASN group was characterized by an enrichment of *A. fabarum*.

## 4 Discussion

### 4.1 The nutritional and fermentation quality of whole-plant corn silage

To achieve suitable nutritional digestibility for animals, the optimal DM content of whole corn silage is 33–35%, corresponding to the 1/3–2/3 milk line stage of harvest (Lin et al., 1992; Liu et al., 2014). In this study, the DM content of the corn material prior to ensiling satisfied the aforementioned criteria. Following a 60-day ensiling period, no significant differences were observed in the DM, NDF, and ADF content between different treatments (*p* < 0.05), this suggests that under well-sealed conditions, different environmental conditions and the inoculation of *A. pasteurianus* did not result in any alterations to the fiber quality of the silage. In the D60 group, the final pH of all treatments was below 3.80, indicating that the corn silage was well-preserved (Cui et al., 2020). This finding is consistent with the observation made by McDonald et al. (1991) that the final pH of corn silage is typically between 3.7 and 4.0, this is likely due to the lower buffering capacity of corn silage, which is in the range of 200–250 mE·kg^−1^ DM. This pH level is recommended for the inhibition of bacterial growth, particularly that of harmful bacteria. The microbial counts indicated that the number of LAB was consistent with the epiphytic microbiota of whole corn silage. The addition of *Acetobacter* bacteria under anaerobic conditions had a negligible effect on the fermentation quality of the silage, as the organic acids produced by the epiphytic LAB primarily reduce the pH (Jatkauskas et al., 2018). The NH_3_-N content of the feed after 60 days of fermentation was significantly lower than that of the feed exposed to aerobic conditions, this may be attributed to the low acidity (pH), which inhibits protein degradation (Basso et al., 2014). It was observed that no yeast growth was evident when the silage was inoculated with acetic bacteria, which may be a principal factor contributing to aerobic spoilage (Spoelstra et al., 1988). Additionally, the study did not find a significant increase in AA content during the fermentation phase, possibly because the conversion of ethanol to acetic acid is often unobservable under practical or laboratory conditions (Honig and Woolford, 1980). The LA and AA content in this study is similar to other corn silages (Jiang et al., 2020).

### 4.2 The aerobic stability of whole-plant corn silage

The utilization of corn silage subsequent to the opening of the bag represented the most critical aspect of the production process. The aerobic and acid-tolerant microorganisms that were activated by exposure to air oxidize different substrates, thereby promoting their growth and reproduction. This process, known as aerobic spoilage, reduced the palatability and feed value of the silage (Weiss et al., 2016). It is commonly accepted that silage exposed to air will undergo an increase in temperature and pH. The occurrence of spoilage was typically defined as a temperature increase in the center of the silage exceeding the ambient temperature by 2°C. The term “aerobic stability” was used to describe the ability of silage to resist spoilage during the transition from anaerobic to aerobic conditions (Drouin et al., 2021). A lack of aerobic stability in silage results in the oxidation and degradation of nutrients, which in turn lead to a reduction in feed intake and digestibility by animals. This, in turn, affected animal growth, reproduction, and milk quality (Oliveira et al., 2017). It is therefore imperative to ensure that corn silage exhibited optimal aerobic stability to guarantee that livestock receive nutrients with minimal loss, in conjunction with an absence of toxins and spores (Kung et al., 2003).

The capacity of silage to withstand deterioration was contingent upon the prevailing environmental conditions and the initial concentration of *A. pasteurianus* present. The application of *A. pasteurianus* exhibited a response to the treatments that was notably temperature-dependent. In addition to exogenously added *Acetobacter* bacteria, there was also the growth of endogenous *Acetobacter* bacteria. Dolci et al. (2011) posited that the proliferation of *Acetobacter* bacteria may result from the presence of excessive yeast or damage caused by propionic acid accumulation. This genus was capable of converting ethanol to acetic acid in the presence of oxygen and was also able to oxidize lactate and acetate to carbon dioxide and water. Consequently, it plays a pivotal role in the triggering of aerobic spoilage during the opening stage (Pahlow and Muck, 2009). Except for Group 7, the aerobic stability of ASN was significantly higher than that of ASA. The addition of aerobic bacteria accelerated the oxidation of acetate, disrupting the strong acidic environment at the end of silage fermentation. This sped up the growth of yeast and mold and hindered the lipophilic action of undissociated acetic acid on saprophytic bacteria, thereby accelerating the aerobic deterioration of silage upon contact with air and shortening the aerobic storage time (Ávila and Carvalho, 2020). The longest aerobic stability duration was observed under the conditions of T20M70A50 in Group 7, indicating that low temperature (20°C), high DM content (30%), and inoculation with a 50% concentration of *A. pasteurianus* bacteria effectively prevent the temperature rise during aerobic storage of corn silage. This result was at odds with the findings of Wilkinson and Davies (2013), which indicated that silage with a high DM content was more susceptible to aerobic spoilage than that with a low DM content. This discrepancy may be attributed to the fact that the preceding study did not take environmental temperature into account. In general, whole-corn silage was more susceptible to aerobic spoilage in hot and humid climates. In a study conducted by Santos et al. (2013), the optimal pH range for silage was identified as 3.80–4.20. In this experiment, Groups 2, 4, 6, 7, 9, 10, 11, 12, 15, and 16 were found to meet the requisite criteria for the production of good silage. In the current experiment, the aerobic stability time of Group 8 in the ASN group was found to be significantly lower than that of Group 2. This discrepancy may be attributed to the higher pH and significantly lower LA and AA content observed in Group 8 (*p* < 0.05). It has been demonstrated that the AA content of silage not only facilitates a reduction in pH but also effectively inhibits the growth of yeast and mold, thereby enhancing the aerobic stability of the silage (Kleinschmit and Kung, 2006).

The application of response surface methodology (RSM) enabled the identification of the factors that contribute to the proliferation of *A. pasteurianus*. The elevated high *F*-value and significantly low *p*-value verified the efficacy of the proposed model in anticipating and responding to temperature, DM content, and initial concentration of *A. pasteurianus* (Oroian et al., 2020). Furthermore, the insignificant lack of fit demonstrates that the model was an accurate representation and can predict the response values with precision (Wu et al., 2023). The low coefficient of variation indicates that the RSM regression model has good predictive accuracy, thereby ensuring that the model was comprehensive, accurate, and reliable.

### 4.3 The microbial community dynamics of whole-plant corn silage

The results demonstrated that the sequence depth in this study is sufficient to yield reliable conclusions. It is widely accepted that the gradual replacement of the complex microbial community of the raw material by LAB following anaerobic fermentation is a key indicator of successful silage fermentation. Consequently, the microbial richness undergoes a marked decline following the completion of the fermentation process. A reduction in bacterial community diversity was observed in the silage material following the fermentation process, in comparison to the original raw material. One potential explanation was that despite the attachment of a limited quantity of *L. plantarum* (1.06%), the production of sufficient lactic acid was unable to reduce the pH and impede the proliferation of other deleterious microorganisms. The ASN group exhibited greater bacterial richness and diversity than the ASA group, potentially due to the proliferation of acid-sensitive spoilage microorganisms during aerobic exposure of the silage feed (Wilkinson and Davies, 2013). Gharechahi et al. (2017) observed that an increase in the abundance of dominant bacteria resulted in a reduction in microbial community diversity. Following aerobic exposure, the relative abundance of *Acetobacter* was observed to dominate all treatment groups, which in turn led to a reduction in bacterial diversity and richness.

A substantial body of research has identified a range of microbial communities and successions in forages subjected to ensiling, both prior to and following the process. It can be concluded that the composition of the microbial community plays a pivotal role in the fermentation of ensiled forage. To gain a comprehensive understanding of the complex process of silage, it is essential to have an in-depth knowledge of the microbial community composition. *Leuconostoc* and *Klebsiella* were common bacteria that were naturally attached to corn in hot and humid regions (Cai, 1999). The results of studies on silage microorganisms indicated that *Proteobacteria* initially dominate in RAW, but were subsequently replaced by *Firmicutes* following the ensilage process. This finding was consistent with the results of the present study (Yang et al., 2019). The relative abundances of *Klebsiella pneumoniae* and *Serratia marcescens* were found to be elevated in RAW, both belonging to the family *Enterobacteriaceae*. Additionally, trace quantities of *Clostridium guangxiense*, a member of the *Clostridiaceae* family, were identified in the D60 group. The presence of *Enterobacteriaceae* and *Clostridiaceae* was undesirable as these bacteria are capable of metabolizing WSC to produce organic acids, thereby competing with LAB for nutrients. This can result in the fermentation of WSC to produce acetic and butyric acids (Lv et al., 2020), which can lead to high DM loss and poor silage quality (Wang et al., 2019). Following a 60-day ensiling period, the abundance of *Firmicutes* was observed to be higher in the D60 group than in the RAW group, with *Lactobacillus* becoming the second dominant genus in D60. *Lactobacillillus* were of great importance in the ensiling process, producing LA, lowering pH, and inhibiting the growth of undesirable bacteria, dominating high-quality silage, which can explain the good fermentation quality of whole-corn silage.

The composition of *A. pasteurianus* exhibited both some similarities and differences between silage samples subjected to disparate treatments at varying stages. It is currently the most prevalent bacterium following aerobic spoilage in the study, potentially due to its extensive distribution (Johnson et al., 1999). The research indicated that *Acetobacter* was hard to find in North America, but was more frequently identified in Asia and Europe, particularly in silage originating from the hot and humid regions of Asia (Oliveira et al., 2017). Previous studies have revealed that the information provided at the genus level was either limited or erroneous. For instance, there was a discrepancy between the roles assigned to homofermentive and heterofermentive LAB in silage, as evidenced by conflicting reports in the literature. Consequently, based on the bacterial abundance at the species level, this experiment identified two species of *Acetobacter* present during the silage and aerobic exposure stages: *A. pasteurianus* and *A. fabarum*. In the absence of inoculated *A. pasteurianus*, a small quantity of *A. fabarum* (0.35%) was also identified in the RAW group. The *Acetobacter* genus comprises a diverse range of species, each with distinct characteristics and functions. For instance, some oxidize ethanol to acetic acid under aerobic conditions (Nanda et al., 2021), while others engage in nitrogen fixation (Fuentes-Ramírez et al., 2001), pigment production (Malimas et al., 2009), or exopolysaccharide production (Gullo et al., 2017). However, *A. fabarum* has never been identified in silage and its role remained unclear. In the D60 group, *A. pasteurianus* emerged as a dominant species, with a relative abundance of 30.31%. This dominance may be attributed to the anaerobic fermentation of *A. pasteurianus* introduced over a short period. During the aerobic exposure stage, the dominance of *L. plantarum* was superseded by that of *A. pasteurianus* as a consequence of the alteration in the environment from anaerobic to aerobic. The abundance of *A. pasteurianus* in ASA was significantly higher than in other groups, which may be attributed to the rapid proliferation of *A. pasteurianus* under aerobic conditions. It is noteworthy that a high abundance of *A. fabarum* was observed in the uninoculated ASN group, in contrast to the ASA treatment group where *A. pasteurianus* was inoculated after 60 days of ensiling. The combined data on aerobic stability revealed that the ASN group exhibited significantly enhanced aerobic stability compared to the ASA group. This indicated that, despite both *A. pasteurianus* and *A. fabarum* belonging to the *Acetobacter* genus, their impact on the aerobic stabilization of silage may be different. The introduction of *A. pasteurianus* resulted in a rapid onset of aerobic destabilization, whereas the presence of *A. fabarum* did not appear to exert a detrimental impact on aerobic stability. The current research literature indicated that *A. fabarum* was commonly found during the fermentation of cocoa beans and moist maize dough (Moens et al., 2014), and was also a main species found in fig vinegar (Sommer and Newell, 2019), *A. fabarum* exhibited a slower rate of ethanol oxidation, with lactic acid undergoing oxidation primarily to acetonitrile and acetic acid. Following the depletion of ethanol, the oxidation of acetic acid commenced (Moens et al., 2014). Additionally, *A. fabarum* has been observed to engage in a mutualistic interaction with *L. brevis* (Sommer and Newell, 2019). However, there was a paucity of research on its role in silage, necessitating further investigation to ascertain its precise function within the silage process.

The LEfSe results demonstrated a notable decline in the epiphytic pathogens on the corn raw material, from the initial stages of processing through to the anaerobic fermentation phase. This was accompanied by a surge in the prevalence of acid-producing bacteria. *Lactobacillus* bacteria play an indispensable role in the conversion of nutrients into volatile fatty acids, thereby improving the quality and aerobic stability of silage (Tahir et al., 2023). Furthermore, research has demonstrated that *L. plantarum*, a species within the *L.* genus, is associated with an elevated acetic acid content in long-term silage (Nishino et al., 2012). This is because the bacterium is capable of metabolizing lactic acid into acetic acid under conditions of low sugar availability (Lindgren et al., 1990). The metabolism of lactic acid may be a contributing factor to the observed enhancement of acetic acid fermentation, as the increase in acetic acid content is significantly greater than the decrease in lactic acid content (Li and Nishino, 2013), *Clostridium guangxiense*, which was isolated from the D60 group, is a high-caproic acid producer, and its primary function is the synthesis of ethyl caproate. It is a strictly anaerobic spore-forming bacterium that can grow in the absence of ethanol or with an ethanol content below 7%, utilizing a variety of substrates (Tracy et al., 2012). Moderate quantities of ethanol can serve as a carbon source for caproic acid bacteria, and at a pH of 6, the acetic acid content is also elevated (Xie and Chen, 2018). The source of volatile fatty acids was inconsequential, they all resulted in a reduction in the pH of silage, thereby extending its shelf life (Larissa et al., 2021).

## 5 Conclusion

The presence of *A. pasteurianus* and *A. fabarum* in whole-corn silage was a natural occurrence in hot and humid areas. During the conventional fermentation stage, *A. pasteurianus* did not negatively impact the quality of whole-corn silage when properly sealed. However, a transition from anaerobic to aerobic conditions resulted in a rapid decline in the aerobic stability of the silage inoculated with *A. pasteurianus*. The presence of *A. fabarum* did not appear to exert a detrimental impact on the aerobic stability of the silage. RSM analysis demonstrated that at a temperature of 28°C, a DM content of 25%, and an initial *A. pasteurianus* concentration of 5.2 × 10^5^ cfu·ml^−1^, the highest concentration of *A. pasteurianus* was observed during the aerobic exposure stage. Future efforts should be focused on the prevention and control of *A. pasteurianus* in whole-plant corn silage to prolong aerobic stability in hot and humid regions.

## Data Availability

The original contributions presented in the study are publicly available. This data can be found here: https://www.ncbi.nlm.nih.gov/, SRA: SRP530196, BioProject: PRJNA1155248.
