# Corrigendum: Microbiome and response surface methodology analyses reveal *Acetobacter pasteurianus* as the core bacteria responsible for aerobic spoilage of corn silage (*Zea mays*) in hot and humid areas

**DOI:** 10.3389/fmicb.2024.1513623

**Published:** 2024-11-15

**Authors:** Rui Bai, Haiping Li, Shiyong Chen, Xianjun Yuan, Youjun Chen, Yanling Huang, Qingping Zhou, Hao Guan

**Affiliations:** ^1^Sichuan Zoige Alpine Wetland Ecosystem National Observation and Research Station, Southwest Minzu University, Chengdu, China; ^2^College of Grassland Resources, Southwest Minzu University, Chengdu, China; ^3^College of Animal Science and Veterinary Medicine, Southwest Minzu University, Chengdu, China; ^4^School of Mathematics and Statistics, Qinghai Normal University, Xining, China; ^5^College of Agro-grassland Science, Nanjing Agricultural University, Nanjing, China

**Keywords:** *Acetobacter pasteurianus*, whole-plant corn silage, aerobic stability, bacterial community, response surface methodology

In the published article, there were several errors in the affiliations. Instead of:

“Rui Bai^1^, Haiping Li^2^, Shiyong Chen^1, 3^, Xianjun Yuan^4^, Youjun Chen^3, 5^, Yanling Huang^1^, Qingping Zhou^3, 5^ and Hao Guan^3, 5*^

^1^College of Animal Science and Veterinary Medicine, Southwest Minzu University, Chengdu, China

^2^School of Mathematics and Statistics, Qinghai Normal University, Xining, China

^3^Sichuan Zoige Alpine Wetland Ecosystem National Observation and Research Station, Southwest Minzu University, Chengdu, China

^4^College of Agro-grassland Science, Nanjing Agricultural University, Nanjing, China

^5^College of Grassland Resources, Southwest Minzu University, Chengdu, China,”

it should be:

“Rui Bai^3^, Haiping Li^1, 2, 4^, Shiyong Chen^1, 3^, Xianjun Yuan^5^, Youjun Chen^1, 2^, Yanling Huang^3^, Qingping Zhou^1, 2^ and Hao Guan^1, 2*^

^1^Sichuan Zoige Alpine Wetland Ecosystem National Observation and Research Station, Southwest Minzu University, Chengdu, China

^2^College of Grassland Resources, Southwest Minzu University, Chengdu, China

^3^College of Animal Science and Veterinary Medicine, Southwest Minzu University, Chengdu, China

^4^School of Mathematics and Statistics, Qinghai Normal University, Xining, China

^5^College of Agro-grassland Science, Nanjing Agricultural University, Nanjing, China.”

The authors apologize for this error and state that this does not change the scientific conclusions of the article in any way. The original article has been updated.

